# Frequent In-Migration and Highly Focal Transmission of Dengue Viruses among Children in Kamphaeng Phet, Thailand

**DOI:** 10.1371/journal.pntd.0001990

**Published:** 2013-01-17

**Authors:** Maia A. Rabaa, Chonticha Klungthong, In-Kyu Yoon, Edward C. Holmes, Piyawan Chinnawirotpisan, Butsaya Thaisomboonsuk, Anon Srikiatkhachorn, Alan L. Rothman, Darunee Tannitisupawong, Jared Aldstadt, Ananda Nisalak, Mammen P. Mammen, Robert V. Gibbons, Timothy P. Endy, Thanyalak Fansiri, Thomas W. Scott, Richard G. Jarman

**Affiliations:** 1 Center for Infectious Disease Dynamics, Department of Biology, The Pennsylvania State University, University Park, Pennsylvania, United States of America; 2 Department of Virology, Armed Forces Research Institute of Medical Sciences, Bangkok, Thailand; 3 Fogarty International Center, National Institutes of Health, Bethesda, Maryland, United States of America; 4 Division of Infectious Diseases and Immunology, Department of Medicine, University of Massachusetts Medical School, Worcester, Massachusetts, United States of America; 5 Institute for Immunology and Informatics, University of Rhode Island, Providence, Rhode Island, United States of America; 6 Department of Geography, University at Buffalo, Buffalo, New York, United States of America; 7 Department of Infectious Diseases, State University of New York at Syracuse, Syracuse, New York, United States of America; 8 Department of Entomology, Armed Forces Research Institute of Medical Sciences, Bangkok, Thailand; 9 Department of Entomology, University of California Davis, Davis, California, United States of America; University of California, Berkeley, United States of Ameruca

## Abstract

Revealing the patterns and determinants of the spread of dengue virus (DENV) at local scales is central to understanding the epidemiology and evolution of this major human pathogen. We performed a phylogenetic analysis of the envelope (E) genes of DENV-1, -2, -3, and -4 isolates (involving 97, 23, 5, and 74 newly collected sequences, respectively) sampled from school-based cohort and village-based cluster studies in Kamphaeng Phet, Thailand, between 2004 and 2007. With these data, we sought to describe the spatial and temporal patterns of DENV spread within a rural population where a future vaccine efficacy trial is planned. Our analysis revealed considerable genetic diversity within the study population, with multiple lineages within each serotype circulating for various lengths of time during the study period. These results suggest that DENV is frequently introduced into both semi-urban and rural areas in Kamphaeng Phet from other populations. In contrast, the persistence of viral lineages across sampling years was observed less frequently. Analysis of phylogenetic clustering indicated that DENV transmission was highly spatially and temporally focal, and that it occurred in homes rather than at school. Overall, the strength of temporal clustering suggests that seasonal bottlenecks in local DENV populations facilitate the invasion and establishment of viruses from outside of the study area, in turn reducing the extent of lineage persistence.

## Author Summary

Long-term cohort studies of dengue virus (DENV), the most common vector-borne viral disease of humans, are essential to understand the epidemiology and evolution of this important human pathogen, and may assist in predicting the evolutionary response to vaccination. We utilized DENV gene sequences and information on the locations and timing of infected children within a primary school-based cohort in Kamphaeng Phet, Thailand to investigate the spatial and temporal relationships among viruses isolated from 2004 to 2007. We found that all four DENV serotypes circulated in the region during the study period, with the presence of multiple viral lineages within each serotype. Viruses sampled closely in time and space were generally very closely related. More genetic variation was observed across districts in a given year and within the same district across different years. The high genetic similarity among viruses during each season and the rare persistence of these lineages through multiple seasons suggest that seasonal reductions in the force of infection through changes in mosquito transmission and fluctuations in human population immunity are key factors shaping the genetic diversity of dengue virus diversity in this region. The importation of DENV by human movement from other populations is therefore an important generator of DENV diversity even in hyperendemic areas.

## Introduction

Dengue is the leading cause of mosquito-borne viral disease worldwide, and dengue fever (DF) and dengue hemorrhagic fever (DHF) continue to increase in both incidence and geographic range. Recent estimates are that over 50 million DENV infections occur each year, including 500,000 hospitalizations for DHF, primarily among children [1], [2]. Dengue viruses (DENV) are single-stranded, positive-sense RNA viruses (family *Flaviviridae*, genus *Flavivirus*) that are comprised of four antigenically distinct serotypes (or viruses; DENV-1, DENV-2, DENV-3, and DENV-4) that co-circulate in many endemic areas in the tropics and sub-tropics. The phenomenon of co-circulation of multiple DENV serotypes is referred to as hyperendemicity and is believed to increase the risk of severe disease in a population [3]–[5]. Hyperendemicity has also complicated vaccine development, and as yet there is no commercially available, licensed vaccine, although a number of vaccine prospects are currently being investigated in clinical trials [6], [7].

An understanding of the spatial and temporal patterns of DENV spread at various scales is central to determining the factors responsible for both the emergence and the persistence of DENV, and may assist in predicting the spread of DENV following imperfect vaccination. Molecular epidemiological studies of global DENV populations play a key role in understanding the mechanisms of DENV evolution by elucidating critical aspects of epidemiological history, including virus population growth and decline, lineage replacement events, patterns of spatial migration, and rates of evolutionary change. The growing data base of full and partial DENV genome sequences has resulted in several detailed phylogeographic studies, but the focus of these has generally been on endemic and epidemic spread in urban and semi-urban areas [8]–[10]. To date, few studies have examined long-term DENV transmission dynamics in endemic rural areas, where epidemiological patterns may differ from those in more densely populated regions due to environment, social factors, and public health infrastructure.

The prospect of vaccine trials and eventual large-scale vaccination to combat dengue warrants an examination of the dynamics of endemic DENV spread at various scales over multiple years. Long-term cohort studies of DENV infection in children in rural Kamphaeng Phet, Thailand, have greatly increased our understanding of the epidemiology and evolution of DENV in an endemic rural area, and provide a unique context to assess DENV transmission dynamics in rural areas [11]–[18]. In this study we examined the spatial and temporal patterns of DENV spread using sequence data from isolates obtained over several years in a longitudinal cohort undergoing school absence-based surveillance and a concurrent geographic cluster study [12], [18]. The objectives of this study were to characterize the genetic diversity of DENV circulating within a rural population and to investigate whether DENV spread was spatially and temporally focal within this area of stable hyperendemic transmission. Characterizing the genetic diversity and patterns of spread in this population is particularly relevant given that Kamphaeng Phet is a planned site for upcoming dengue vaccine trials.

## Materials and Methods

### Study area and selection of schools

The study protocol was approved by the Institutional Review Boards of the Thai Ministry of Public Health (MOPH), Walter Reed Army Institute of Research (WRAIR), University of Massachusetts Medical School (UMMS), University of California at Davis (UCD) and San Diego State University (SDSU). Written informed consent and assent were obtained from all study participants and/or their parents.

The study area and design have been described previously [11], [12], [18]. The epidemiology of dengue is well characterized in this region of Thailand due to long-term cohort studies and surveillance conducted since the 1980s by the Armed Forces Research Institute of Medical Sciences (AFRIMS), a joint research endeavor of the U.S. Armed Forces and the Royal Thai Army Medical Departments. In brief, the study was conducted in Muang District, Kamphaeng Phet, north-central Thailand, a relatively sparsely populated region of Thailand with 233,033 residents in ∼63,500 housing structures located over 1962 km^2^. DENV transmission in this region peaks during an annual ‘dengue season’ from June to November [16].

Eleven primary schools were selected for participation based on the presence of dengue cases among their students in the previous five years, proximity to the AFRIMS field station (including road access), and interest of the school administrators. Selected schools were associated with 32 villages (8445 houses). During the first half of the study, twenty of these villages (4685 houses) were selected for inclusion in the cluster study based on the density of houses, favoring those with houses in close proximity to one another (<100 m). During the second half of the study, all houses within the villages were mapped and used during cluster investigations. Unique codes were assigned for each of the 8445 houses and the associated spatial coordinates and demographics of residents were entered into a Geographic Information Systems (GIS) database [MapInfo (2000) version 6•0; MapInfo Corporation, Troy, New York].

### Selection of school children and village clusters

Primary school children in kindergarten through grade six were followed by active school absence-based surveillance during June to November of each year. An acute blood sample was drawn from cohort subjects who reported a fever in the previous seven days or who had a measured temperature ≥38°C. A convalescent blood sample was drawn 14 days later along with an evaluation of symptoms. Details on study design and overall results have been published previously [12], [18]. Acute blood samples underwent testing, including semi-nested reverse transcriptase polymerase chain reaction (RT-PCR) for detection of DENV RNA according to the protocol of Lanciotti et al. [19] with the following modifications. Avian myeloblastosis virus (AMV RT, Promega, Madison, WI) reverse transcriptase was used in the first round RT-PCR. The concentrations of primers used in the RT-PCR and nested reactions were reduced from 50 pmol to 12.5 pmol per reaction and the nested PCR amplification cycles were increased to 25.

Cohort subjects who were dengue PCR-positive and negative from an acute blood sample drawn within three days of illness onset served as an “index” case for a positive cluster investigation; non-dengue acutely ill subjects served as index cases for negative control clusters. In each cluster, ten to 25 children aged six months to 15 years living within a 100-meter radius of the index case were enrolled regardless of symptomatology. These contact subjects were evaluated at days 0, 5, 10, and 15 with temperature measurement and a symptom questionnaire covering the previous five days. Blood samples were collected on days 0 and 15 and tested for dengue by RT-PCR; virus isolation was attempted in C6/36 cells from all cohort and cluster dengue PCR-positive samples.

### Entomologic evaluations

Female adult *Ae. aegypti* were collected, after obtaining written permission from the residents, using backpack aspirators from inside and immediately surrounding each home within the cluster, [12], [18]. Female *Ae. aegypti* were screened for DENV by RT-PCR using a modified protocol [20]. In brief, pools of ten mosquitoes were made by combining 14 µl from individual mosquito suspensions (in 100 µl of RPMI containing 1% L-glutamine and 10% heat-inactivated FBS) and clarified by centrifugation at 8000 rpm at 4°C for 20 min. From positive pools, individual mosquitoes were assayed by serotype-specific PCR using 14 µl of the original suspension in 126 µl of diluent. DENV from individual mosquitoes were amplified by intrathoracic inoculation in *Toxorhynchites splendens* mosquitoes and/or by passaging three times in C6/36 cells.

### Virus isolation and sequencing

For human DENV PCR-positive samples, virus isolation was performed in C6/36 cells and/or *Toxorhynchites splendens* mosquitoes as previously described [21], [22]. Viral RNA was prepared for sequencing from 140 µl of human serum, mosquito suspension, or culture fluid using the QIAamp viral RNA mini kit (QIAGEN, Germany) according to the manufacturer's instructions. RT-PCR was performed using random hexamer oligonucleotides with the SuperScript first-strand synthesis system (Invitrogen) according to the manufacturer's instructions. Sequencing of all PCR-positive, isolation-positive samples was attempted but insufficient RNA and isolation failures resulted in some PCR positive cohort and cluster samples to be excluded in this study. The DNA fragments of the envelope gene region of 97 DENV-1, 23 DENV-2, 5 DENV-3, and 74 DENV-4 were amplified by PCR using 5 µl of cDNA in a 50 µl reaction mixture containing 0.3 mM dNTPs, 2.5 U AmpliTaq DNA polymerase (Applied Biosystems), 1× PCR buffer, 1.5 mM MgCl_2_ and 15 pmol of each forward and reverse primer. PCR reactions for DENV-1, -3, and 4 were subjected to 1 cycle of 95°C for 5 min, 35 cycles of 94°C for 30 sec, 50°C for 1 min, 72°C for 2 min, and 1 cycle of 72°C for 15 min. PCR reactions for DENV-2 were subjected to the same thermal conditions as the others, except the annealing temperature was changed to 55°C. The PCR-amplified DNA fragments were purified using QIAquick PCR purification kits (QIAGEN) according to the manufacturer's instructions. Purified DNA fragments were used for sequencing.

Sequencing reactions were performed using the DYEnamic ET Dye Terminator sequencing kit (GE Healthcare Bio-Sciences) according to the manufacturer's instructions. Sequencing primers are available upon request. Sequencing products were cleaned by standard precipitation prior to sequencing in a MegaBACE 500 automated DNA sequencer (GE Healthcare Bio-Sciences). Overlapping nucleic acid sequences were combined for analysis and edited using SEQUENCHER software (Gene Code Corporation).

### Phylogenetic analysis

A total of 97 DENV-1, 23 DENV-2, 5 DENV-3, and 74 DENV-4 E gene sequences were obtained from blood samples of infected children included in the school-based cohort and from mosquitoes and infected children detected in village clusters in Kamphaeng Phet from 2004 to 2007 (Table 1). School-based surveillance accounted for the majority of DENV isolated in this study; 95 were not associated with cluster investigations and 47 served as cluster index cases (Table 2). From 50 positive and 53 negative cluster investigations initiated during the study period, 47 index case dengue sequences were available and19 clusters yielded at least one DENV E gene sequence from a village contact, while eight clusters yielded sequences obtained exclusively from local mosquitoes. There were an additional four sequences obtained from three negative clusters.

**Table 1:** DENV sequences from school children and community clusters in five sub-districts of KPP, 2004–2007.

Year	Serotype	Number of sequences	Number of subdistricts	Number of villages
**2004**	DENV-1	0	0	0
	DENV-2	7	3	4
	DENV-3	1	1	1
	DENV-4	24	5	14
**2005**	DENV-1	2	2	2
	DENV-2	3	3	3
	DENV-3	4	1	1
	DENV-4	15	3	8
**2006**	DENV-1	59	5	16
	DENV-2	2	2	2
	DENV-3	0	0	0
	DENV-4	33	4	10
**2007**	DENV-1	36	5	16
	DENV-2	11	3	5
	DENV-3	0	0	0
	DENV-4	2	1	2

**Table 2:** Number of DENV sequences used in this study.

Serotype	School surveillance	Index case initiating village cluster investigation*	Village contact of index case	Mosquito
**DENV-1**	38	25	24	10
**DENV-2**	12	6	2	3
**DENV-3**	1	1	1	2
**DENV-4**	34	15	18	1

* All samples were obtained via school absence-based surveillance within a childhood cohort and village cluster studies activated by illness within the cohort in Kamphaeng Phet, Thailand from 2004–2007. Index cases were detected through school-based surveillance and subsequently selected for village cluster investigation. These are not included in school surveillance counts.

To place these isolates within the context of global DENV evolution, complete E gene sequences of all four serotypes with known date and country of sampling were collected from GenBank. Sequences were manually aligned using Se-AL v2.0a11 (available from http://tree.bio.ed.ac.uk/software/), and initial trees were inferred in PAUP using a neighbor-joining algorithm using the HKY85 model of nucleotide substitution [23]. This enabled us to obtain a provisional estimate of the pattern of genetic diversity of DENV isolated within the study. All study isolates from each serotype were found to be of a single, predominantly Asian genotype (DENV-1, genotype I; DENV-2, Asian I genotype; DENV-3, genotype II; DENV-4, genotype I; see Results). Consequently, global sequence data sets were sub-sampled to include only a subset of closely related sequences of Asian origin from each of the detected genotypes: 1161 DENV-1 (1485 nt), 531 DENV-2 (1485 nt), 304 DENV-3 (1479 nt), and 166 DENV-4 (1485 nt) E gene sequences.

Maximum likelihood (ML) phylogenetic trees of each genotype were then estimated using PAUP [23], utilizing the GTR+I+Γ_4_ model of nucleotide substitution, which was determined by ModelTest [24] to be the best-fit to the data in hand, and employing tree bisection-reconnection (TBR) branch swapping. Bootstrap resampling (1000 replicate neighbor-joining trees under the substitution model described above) was performed to assess phylogenetic support for individual nodes.

### Analysis of spatial and temporal structure

To determine the extent of spatial and temporal structure of DENV within the study area, Bayesian Maximum Clade Credibility (MCC) phylogenetic trees were inferred for the E gene sequences of DENV-1, DENV-2, and DENV-4 collected in Kamphaeng Phet during 2004–2007 using a Bayesian Markov Chain Monte Carlo (MCMC) method implemented in the BEAST package (v1.6.1) [25]. The small number of DENV-3 sequences collected during the study precluded their inclusion in this analysis. A strict molecular clock, a TN93+Γ_4_ model of nucleotide substitution (determined by ModelTest [24] to be the best-fit model of nucleotide substitution for the Kamphaeng Phet-specific DENV data sets) with two codon position divisions (1+2, 3), and a constant population size model were used for all analyses. Mixing under more complex evolutionary and demographic models (including a Bayesian skyline plot model) was poor, and Bayes Factor tests indicated this model to be the most appropriate for each of the four serotypes independently.

We used the BaTS (Bayesian Tip-association Significance testing) program to assess the extent of spatial and temporal structure among DENV populations in Kamphaeng Phet from the posterior samples of trees returned by the BEAST analysis described above [25], [26]. The BaTS program outputs an Association Index (AI) and a Parsimony Score (PS), for which 0 indicates complete population subdivision and 1 suggests random mixing (panmixis), as well as a Maximum Clade (MC) score for each character state (location, school, year, etc.) that indicates the extent of clustering for that state compared to all others. Sequences were coded and tested for clustering by; (i) home sub-district (5 sub-districts; KNT - Kon Tee, NBK - Na Bo Kham, NKC - Nakon Chum, NPL - Nong Pling, TNK - Thep Na Korn), (ii) home village (4 to 9 per district), (iii) school (11 primary schools included in the cohort study and school-based surveillance, isolates from 12 additional schools obtained in the cluster study), (iv) age (0–4, 5–9, to 10–15 years), (v) sex, (vi) clinical syndrome of the patient (includes non-hospitalized DF, hospitalized DF, hospitalized DHFII, hospitalized DHFIII, unknown syndrome) and (vii) year of sampling. For age, sex, and clinical syndrome, mosquitoes were included as a separate group, and unknown clinical traits were recorded as NA. In the case of the school-based analysis, two coding schemes were utilized: (1) subjects with no school listed on file (primarily children younger than school-age) and viruses isolated from mosquitoes were coded as NA and mosquito, respectively, and (2) mosquitoes and subjects associated with no school were coded with the school of the index case of the cluster in which they were identified. Results using scheme 2 are reported here. Results were generally similar, but weaker, using scheme 1.

The cluster design of the study involved focal sampling over very narrow intervals of time and space and, therefore, had the potential to bias the results of BaTS analysis. To ensure that this study design was not adversely affecting our inference of spatial and temporal patterns, Bayesian MCMC trees were also inferred for DENV-1 and DENV-4 isolates obtained only from index cases and through school-based surveillance. Of the isolates obtained through school-based surveillance and not treated as index cases in the cluster study, only a single sequence from each school per year was utilized to account for potential over- or under-sampling among schools. Phylogeny-trait association tests were then performed on a subset of 37 DENV-1 sequences and 28 DENV-4 sequences, considering the home sub-district, village, school of subjects, and year of sampling. Too few sequences were available to perform this analysis on the DENV-2 data set after subsampling.

### Nucleotide sequence accession numbers

DENV E-gene sequences were deposited in GenBank with the following accession numbers: DENV-1 JQ993108-JQ993204, DENV-2 JQ993205-JQ993227 DENV-3 JQ993228-JQ993232 DENV-4 JQ993233-JQ993306.

## Results and Discussion

### Microevolution of DENV in Kamphaeng Phet over four dengue seasons

**Figure 1:**
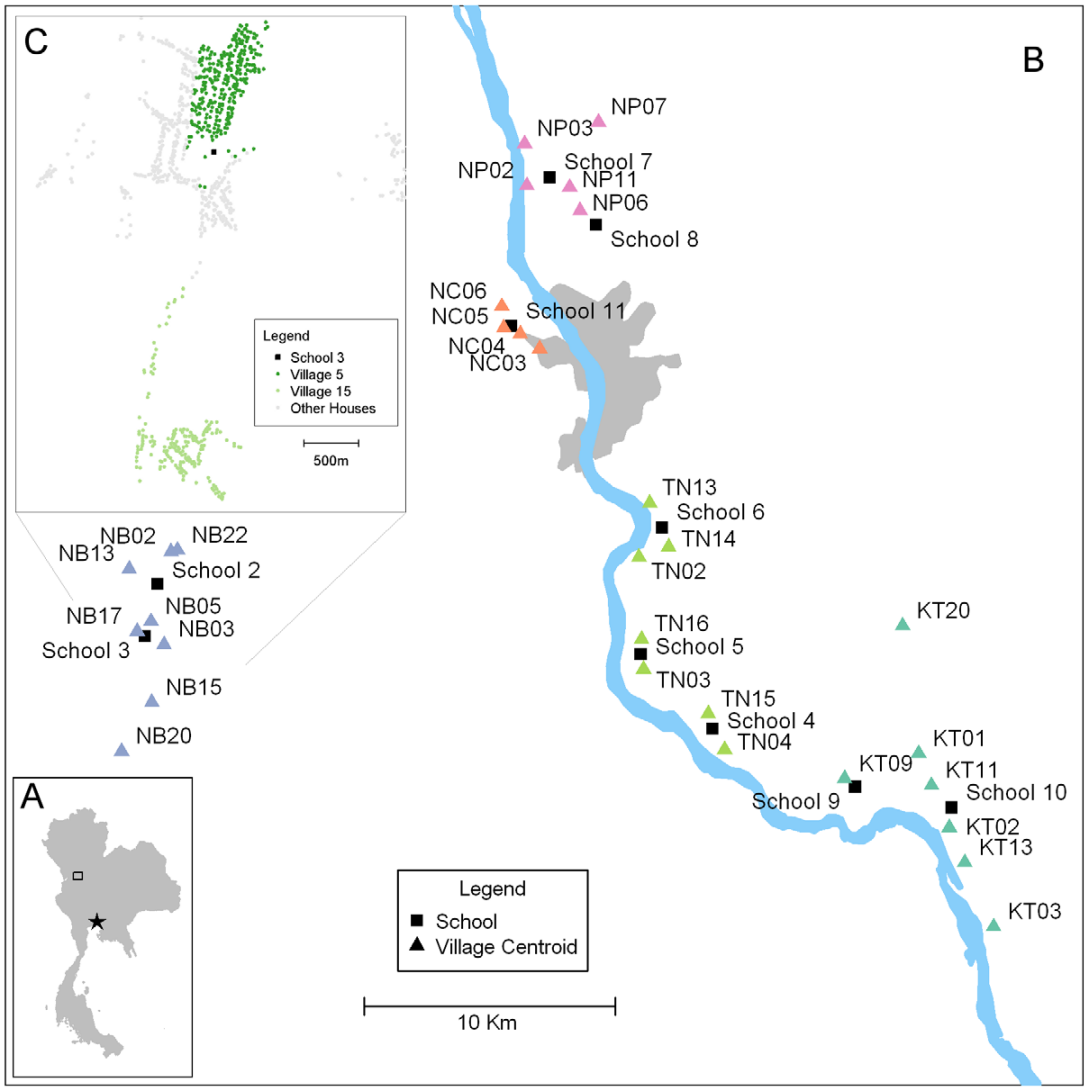
Study location map. **A**: Kamphaeng Phet (box), Thailand is located approximately 300 km Northwest of Bangkok (star). **B**: Map of study area in Muang District, Kamphaeng Phet, Thailand. The locations participating schools are indicated by black boxes and the villages that feed the schools are indicated with triangles. All houses within the villages in this study were GIS mapped, KT, Kon Tee; NB, Na Bo Kham; NC, Nakon Chum; NP, Nong Pling; TN, Thep Na Korn. **C**: A representational GIS mapping of two villages (5 and 15) to indicate the varying density of houses with the villages.

**Figure 2:**
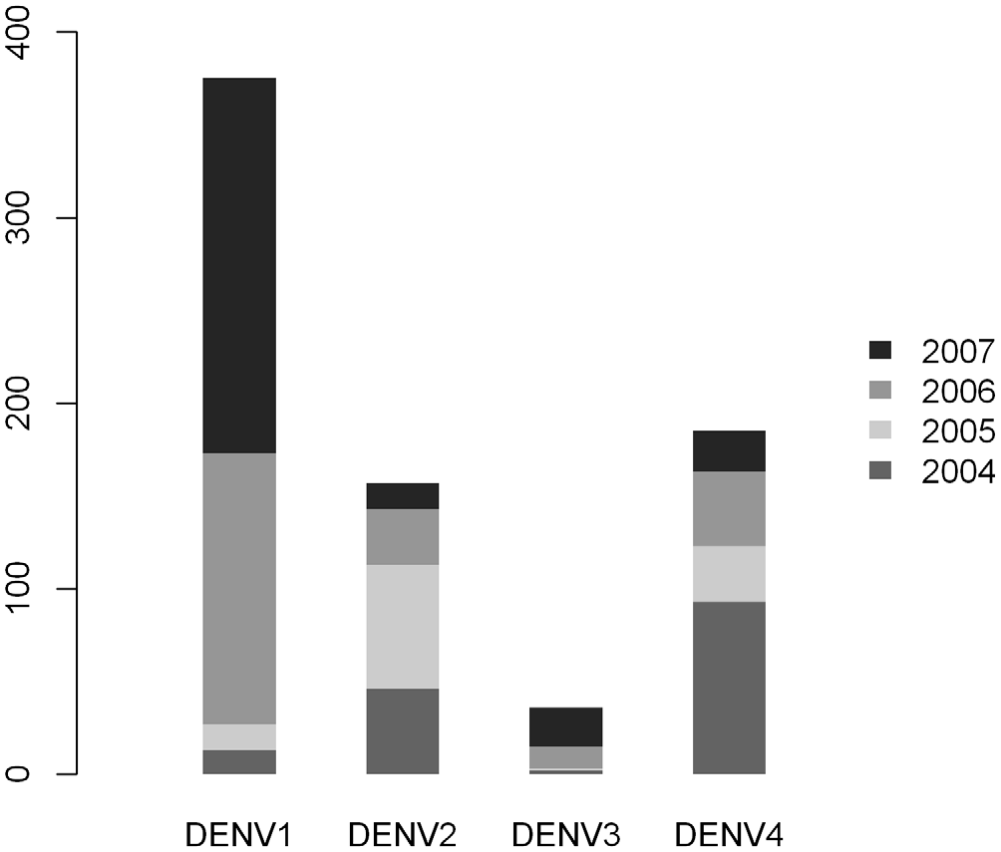
KPPPH data of all reported and PCR confirmed dengue cases in Muang District from 2004–2007. Data collected from this study are included.

To investigate the genetic diversity and structure of DENV populations circulating in Kamphaeng Phet (Figure 1) from 2004 to 2007, we sequenced and analyzed the E genes of viruses collected during school-based surveillance and geographically-based community cluster studies. At least three serotypes circulated in our study population during each year of the study, with all four serotypes detected only in 2005 (Table 1). This pattern generally reflects the relative proportions of DENV isolated through passive surveillance across the region during the period of sampling, although passive surveillance data from the Kamphaeng Phet Provincial Hospital detected all four serotypes circulating each year (Figure 2). Years in which a given serotype was not detected in our study population corresponded to instances in which that serotype was detected at less than 10% frequency during passive surveillance. This suggests that our sampling and sequencing protocol adequately captures the diversity of the viral population circulating in KPP in a given year.

Only a single genotype was represented for each serotype in this data set (DENV-1, genotype I; DENV-2, Asian I genotype; DENV-3, genotype II; DENV-4, genotype I). These four genotypes have been the dominant circulating genotypes in Thailand and much of mainland Southeast Asia for several years, and were all present among previous Thai E gene sequences from Thailand dating back to 2001 and earlier. Although other genotypes were detected in Thailand prior to our study, only DENV-1 genotype I, DENV-2 Asian I genotype, and DENV-3 genotype II were isolated in a study performed in Kamphaeng Phet in 2001; no DENV-4 sequences were isolated during the 2001 study [13]. Thus, it appears that these were the dominant circulating genotypes in Kamphaeng Phet, at least through 2007.

**Figure 3:**
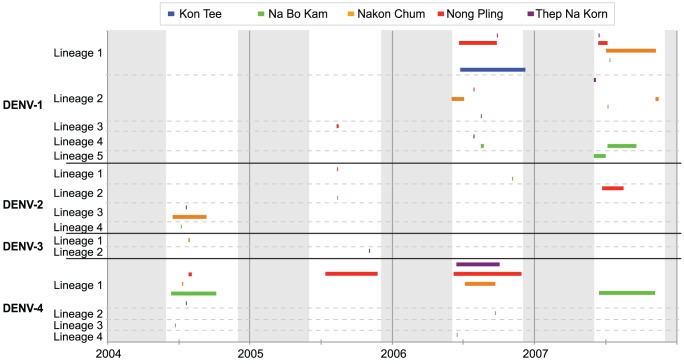
Timing of DENV lineages circulating in five participating sub-districts of KPP, Thailand from 2004–2007. Horizontal bars indicate the time period in which specified lineages were isolated during school-based surveillance (conducted from June to November). Vertical grey bars indicate periods of the year in which cohort sampling was inactive (December to May).

**Figure 4:**
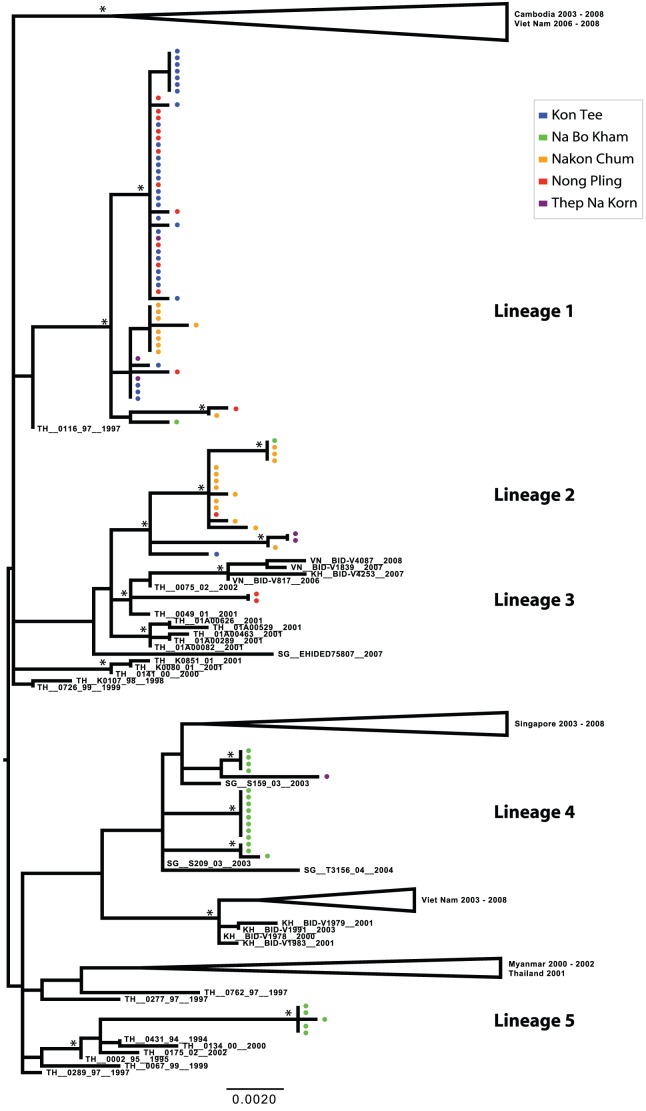
ML tree of representative E gene sequences of DENV-1 genotype I. Districts of Kamphaeng Phet are designated by color (Kon Tee, blue; Na Bo Kham, green; Nakon Chum, orange; Nong Pling, red; Thep Na Korn, purple). Bootstrap support values ≥85% are indicated by an asterisk next to the node. The tree is midpoint rooted for purposes of clarity.

Phylogenetic analysis revealed considerable viral genetic diversity within genotypes in the Kamphaeng Phet region during this period, demonstrated by the presence of phylogenetically distinct lineages within each serotype. Among the DENV-1 isolates detected, five major lineages were present, suggestive of at least five separate virus introductions prior to or during the sampling period (Figures 3 and 4). One lineage was detected briefly in 2005, but was not observed in the following years. Three lineages circulated in 2006, the year in which DENV-1 incidence greatly increased in the region (Figure 2). All of these lineages persisted into the 2007 season, during which one additional lineage was detected. With the exception of Lineage 4, these viral populations were most closely related to viruses isolated in Thailand in the previous decade. Lineage 4 may have been a recent introduction from Singapore (the most closely related geographical region on the phylogenetic tree) that invaded Na Bo Kham sub-district and persisted within that population for at least two years. In all of the lineages except Lineage 2, viruses commonly mixed among sub-districts within a single season.

**Figure 5:**
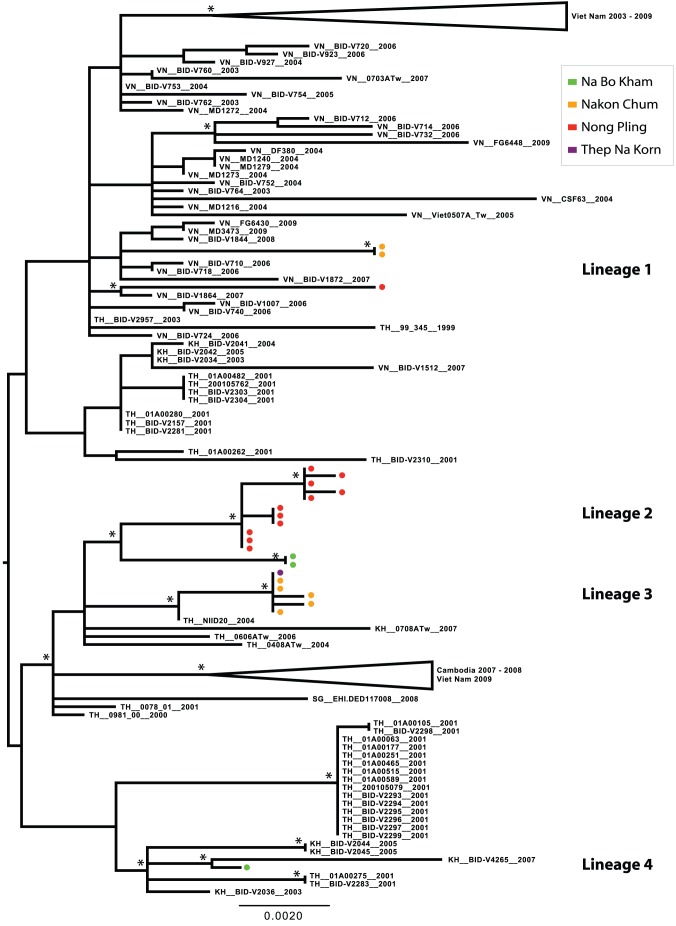
ML tree of representative E gene sequences of the Asian I genotype of DENV-2. Districts of Kamphaeng Phet are designated by color (Kon Tee, blue; Na Bo Kham, green; Nakon Chum, orange; Nong Pling, red; Thep Na Korn, purple). Bootstrap support values ≥85% are indicated by an asterisk next to the node. The tree is midpoint rooted for purposes of clarity.

Despite the small number of sequences obtained, the DENV-2 population also showed considerable genetic diversity. Four lineages were detected between 2004 and 2007, with little mixing detected among sub-districts within a given season (Figures 3 and 5). Interestingly, however, Lineage 2 was first detected in rural Na Bo Kham in 2005, and was not isolated again until in 2007 in the most cosmopolitan of our populations, Nong Pling. This suggests that in this environment viral populations may sometimes move from rural to more densely populated areas rather than the opposite, as has been reported for other locations [8], [9].

**Figure 6:**
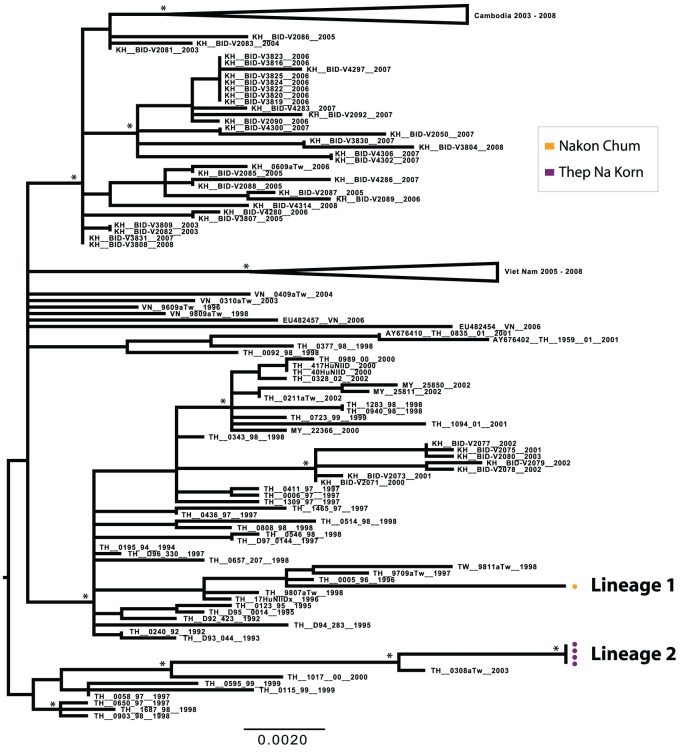
ML tree of representative E gene sequences of DENV-3 genotype II. Districts of Kamphaeng Phet are designated by color (Kon Tee, blue; Na Bo Kham, green; Nakon Chum, orange; Nong Pling, red; Thep Na Korn, purple). Bootstrap support values ≥85% are indicated by an asterisk next to the node. The tree is midpoint rooted for purposes of clarity.

Although DENV-3 exhibited the lowest incidence in the study population during this time period, one lineage was detected in 2004 and a different lineage was detected in 2005 (Figures 3 and 6). Both of these were derived from lineages that were circulating in Thailand in the previous decade, which suggests that rare lineages may persist in rural populations even when disease incidence is low.

**Figure 7:**
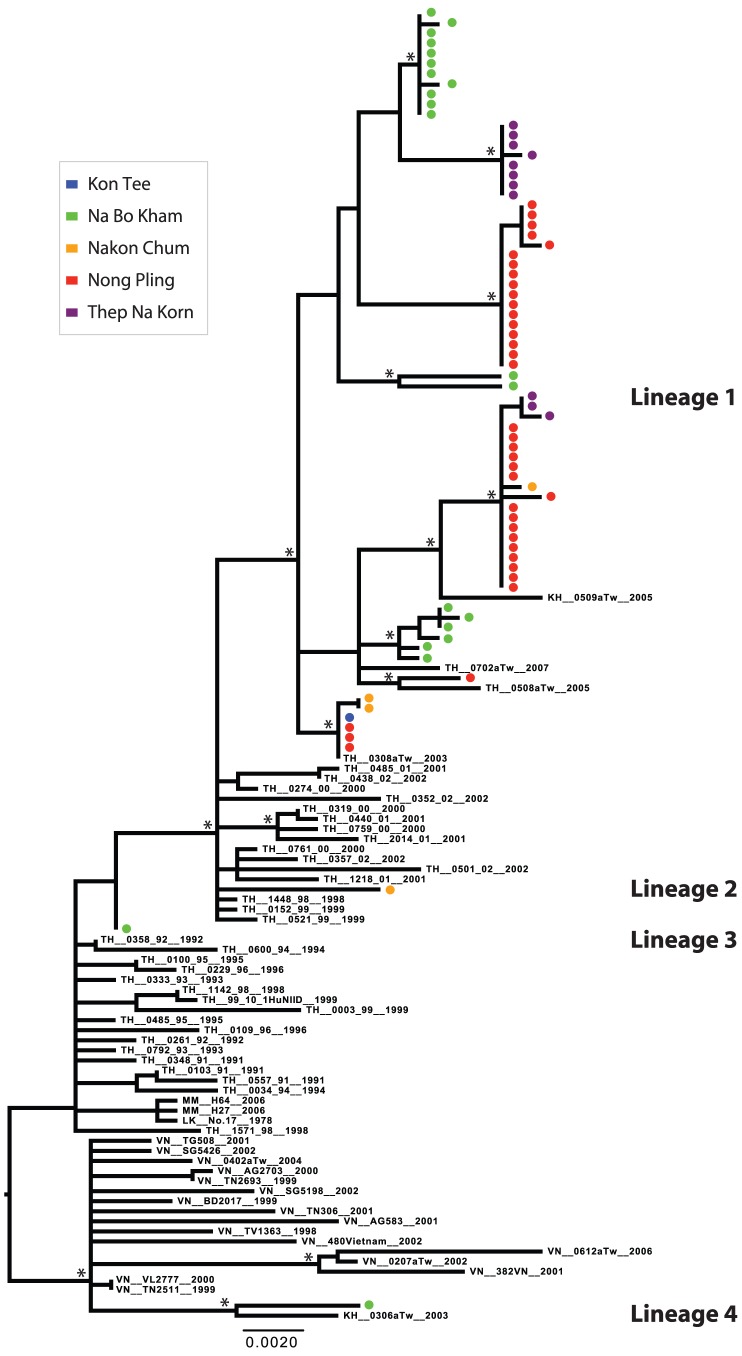
ML tree of representative E gene sequences of DENV-4 genotype I. Districts of Kamphaeng Phet are designated by color (Kon Tee, blue; Na Bo Kham, green; Nakon Chum, orange; Nong Pling, red; Thep Na Korn, purple). Bootstrap support values ≥85% are indicated by an asterisk next to the node. The tree is midpoint rooted for purposes of clarity.

DENV-4 was the dominant serotype detected in Kamphaeng Phet during the first two years of this study. Two lineages were present in 2004; one was represented by a single isolate detected in Na Bo Kham (Lineage 3), and the other was represented by three distinct, primarily sub-district-specific clusters, showing limited mixing in a given year (Lineage 1) (Figures 3 and 7). Lineage 1 appeared to persist as the dominant DENV-4 lineage in the population throughout the study, although multiple distinct populations were present through 2007. Two additional introductions were observed in the Na Bo Kham and Nakon Chum sub-districts in 2006 (Lineage 4 and Lineage 2, respectively). Based on the sequences in this study, these introductions did not appear to result in extended transmission in the region. Interestingly, while most lineages detected appeared to have entered following long-term transmission within Thailand, a single sequence isolated in Na Bo Kham in 2006 (Lineage 4) may have entered through the re-introduction of a Thai lineage following extended transmission elsewhere in Southeast Asia.

It is interesting that many of recent introduction events occurred in Na Bo Kham sub-district; this is the most rural of the populations studied and more distantly positioned and isolated relative to the other study populations. A previous study performed in Kamphaeng Phet during a single, high incidence dengue season detected the greatest genetic diversity among viruses in the most densely populated areas [13], while the current study indicates the opposite. Because most of these lineages did not appear to immediately move into the greater population of Kamphaeng Phet, it is possible that the DENV populations present in Na Bo Kham differ greatly from the rest of the area because there is less mixing with the other populations we sampled. Alternatively, Na Bo Kham may depend on a different population center through which viral variants are introduced. Differences among DENV from Na Bo Kham and the rest of the sub-districts studied indicate that geographically distinct patterns of human movement are important processes in the structure and dynamics of DENV populations [27].

### Spatial and temporal clustering of DENV in Kamphaeng Phet

Among village clusters initiated by dengue cases detected in the school-based cohort, nearly all of the DENV viruses sequenced from both humans and mosquitoes within a cluster during the 14-day period following the initiation of sampling exhibited identical or nearly identical E gene sequences to the index case of their respective clusters. All but three of these sampling clusters involved viruses originating from the same lineage within a single serotype. The three exceptions are described here. First, in the case of DENV-1, one cluster of three samples included an index case and one village contact that possessed identical E genes in Lineage 2 and a second village contact that was infected with a virus in Lineage 1, which was found to be circulating simultaneously in multiple sub-districts during the 2006 and 2007 seasons. Second, a single DENV-1 virus was isolated from a cluster investigation in which the index case and all other village contacts were infected with DENV-4. This DENV-1 isolate fell within Lineage 1 and was the first virus collected within a large phylogenetic cluster of identical E gene sequences isolated in the same village for the following seven to 31 days, from elsewhere in Nong Pling sub-district on days 62 and 97 after the first isolation, and from Thep Na Korn and Kon Tee sub-districts in the following 97 and nine to 108 days, respectively, spanning 2006 to 2007. Third, among DENV-4 isolates, one cluster in Na Bo Kham in 2004 (Lineage 1) included a virus of a divergent lineage (Lineage 3). Multiple closely related viruses were isolated from both humans and mosquitoes, forming a well-supported clade with other viruses from the same sub-district in the same season. A single mosquito was found to be infected with a divergent virus, comprising the only isolate of DENV-4 Lineage 3 detected in this study. That this lineage was only detected in a mosquito and we did not detect evidence of further transmission within the study population, as well as the number of lineages represented by a single isolate in the data set, suggests that multiple DENV lineages commonly enter this population in a single season, some of which fade out without detection, or may persist at low levels with little to no detection in the human population.

Both the sequence data presented here and the epidemiological data collected from the cohort and cluster studies [12] suggest that DENV transmission is indeed highly spatially and temporally focal rather than occurring via simultaneous circulation and mixing of multiple DENV lineages across the region [18]. For confirmation, we performed a statistical analysis of the strength of phylogenetic clustering of viruses sampled closely in space and time. At all levels of spatial aggregation (sub-district, village, school, and sampling cluster) and for all three serotypes investigated (DENV-1, DENV-2, and DENV-4), we detected a significant relationship between phylogeny and space; i.e., there was more clustering by spatial variable than expected by chance alone (Table 3). Similarly, we found a strong phylogenetic clustering of viruses by year of sampling. Hence, we conclude that viral genetic diversity in this population tends to turnover on an annual basis, although lineages may occasionally persist over multiple seasons.

**Table 3:** Phylogeny-trait association tests of phylogenetic structure of geographic, temporal, and clinical traits of DENV infections.

Serotype	Trait	Number of groups	Association Index^1^, ratio of observed to expected (95% CI)	Parsimony Score^1^, ratio of observed to expected (95% CI)	*P*-value^2^
**DENV-1**	**Sub-district**	5	0.31 (0.20, 0.44)	0.44 (0.38, 0.50)	<0.01, <0.01
	**Village**	22	0.50 (0.39, 0.62)	0.61 (0.55, 0.66)	<0.01, <0.01
	**School**	15	0.62 (0.49, 0.77)	0.65 (0.60, 0.70)	<0.01, <0.01
	**Cluster**	31	0.67 (0.53, 0.83)	0.77 (0.71, 0.82)	<0.01, <0.01
	Sex*	3	0.96 (0.70, 1.27)	1.00 (0.84, 1.16)	0.28, 0.54
	Age*	4	1.06 (0.81, 1.38)	1.03 (0.91, 1.16)	0.86, 0.87
	Clinical syndrome*	5	1.01 (0.77, 1.28)	0.99 (0.92, 1.08)	0.57, 0.51
	**Year**	3	0.04 (0.02, 0.11)	0.25 (0.21, 0.32)	<0.01, <0.01
**DENV-2**	**Sub-district**	4	0.19 (0.04, 0.38)	0.51 (0.47, 0.63)	<0.01, <0.01
	**Village**	7	0.28 (0.11, 0.51)	0.59 (0.49, 0.68)	<0.01, <0.01
	**School**	7	0.45 (0.18, 0.84)	0.64 (0.52, 0.80)	0.01, <0.01
	**Cluster**	7	0.34 (0.13, 0.63)	0.68 (0.55, 0.81)	<0.01, <0.01
	Sex*	3	1.08 (0.69, 1.67)	1.07 (0.93, 1.32)	0.71, 0.57
	Age*	4	1.03 (0.67, 1.66)	1.00 (0.81, 1.28)	0.58, 0.36
	**Clinical syndrome** *	5	0.65 (0.39, 1.03)	0.78 (0.67, 0.91)	0.01, 0.01
	**Year**	4	0.05 (0.00, 0.10)	0.37 (0.26, 0.43)	<0.01, <0.01
**DENV-4**	**Sub-district**	5	0.09 (0.02, 0.17)	0.30 (0.26, 0.33)	<0.01, <0.01
	**Village**	19	0.45 (0.32, 0.60)	0.55 (0.49, 0.60)	<0.01, <0.01
	**School**	18	0.53 (0.40, 0.68)	0.63 (0.57, 0.70)	<0.01, <0.01
	**Cluster**	18	0.57 (0.40, 0.76)	0.68 (0.59, 0.76)	<0.01, <0.01
	Sex*	3	0.93 (0.64, 1.31)	0.97 (0.82, 1.17)	0.16, 0.38
	Age*	4	0.95 (0.69, 1.28)	0.95 (0.83, 1.11)	0.21, 0.2
	Clinical syndrome*	5	0.94 (0.64, 1.31)	0.97 (0.91, 1.02)	0.23, 0.14
	**Year**	4	0.02 (0.00, 0.09)	0.29 (0.25, 0.35)	<0.01, <0.01

Traits showing significant phylogenetic structure are indicated in bold.

* Analysis included separate groups for mosquito isolates or unknown traits where applicable.

1 AI and PS scores are based on mean values representing the association between trait and phylogeny.

2 *P*-values are based on median values representing the association between trait and phylogeny.

Among the levels of spatial aggregation analyzed, the home sub-district and village of subjects showed stronger phylogenetic clustering (sub-district association index (AI): 0.09 to 0.31, parsimony score (PS): 0.3 to 0.51; village AI: 0.28 to 0.5, PS: 0.55 to 0.61) for all three serotypes than did the school (AI: 0.45 to 0.62, PS: 0.63 to 0.65) and sampling clusters (AI: 0.34 to 0.67, PS: 0.68 to 0.77) (Table 3). This result is not unexpected because epidemiological data suggest that DENV transmission in this region primarily occurs at a person's home or the home of a friend or relative [12], [18]. Our virological data, therefore, support these earlier results and indicate that these trends continued over the four-year time period. No significant phylogenetic clustering of DENV was observed for age, sex, or clinical syndrome, except in the case of DENV-2, for which relatively weak phylogenetic structure was detected according to clinical syndrome (Table 3). This result was strongly influenced, however, by the existence of two clusters of unknown syndrome and non-hospitalized DF, as well as, by the small number of DENV-2 sequences isolated in this study. It does not appear to result from differences in virulence among viral populations. The use of coding scheme 1, in which subjects associated with no school and mosquitoes were coded as NA and mosquito, respectively, produced weaker associations in the school-based analysis than when these were coded according to the school of the respective index case (data not shown). Specifically, these sequences rarely showed clustering, but instead interrupted or reduced the size of school-based clusters.

By restricting our analysis to those sequences obtained from index cases and through school-based surveillance, we were able to further assess whether highly focal sampling in the village-based cluster design was a primary factor influencing the spatial and temporal structure detected. Results of these analyses were generally similar to those obtained using the full set of sequences (Table 4). Significant clustering was observed at the sub-district, village, school and 100-meter radius cluster levels and within years for both DENV-1 and DENV-4. The strength of village-level clustering was reduced, however, relative to that of sub-districts and schools (Table 4). Further, the diversity within this subsample was comparable to that observed in the full data set, with the loss of only a single lineage (DENV-4 Lineage 3) in subsampling. These results indicate that distinct viral populations may be present in areas separated by only a few kilometers, and suggest that school-based virological surveillance alone captures much of the genetic diversity of DENV within a given area. As such, school-based surveillance may be a practical and efficient indicator of DENV circulating in communities in endemic areas.

**Table 4:** Phylogeny-trait association tests of phylogenetic structure of geographic and temporal traits of DENV-1 and DENV-4.

Serotype	Trait	Number of groups	Association Index^1^, ratio of observed to expected (95% CI)	Parsimony Score^1^, ratio of observed to expected (95% CI)	*P*-value^2^
**DENV-1**	**Sub-district**	5	0.38 (0.21, 0.61)	0.61 (0.51, 0.72)	<0.01, <0.01
	**Village**	20	0.66 (0.49, 0.85)	0.79 (0.71, 0.87)	<0.01, <0.01
	**School**	9	0.46 (0.29, 0.65)	0.67 (0.59, 0.76)	<0.01, <0.01
	**Year**	3	0.16 (0.03, 0.4)	0.43 (0.33, 0.55)	<0.01, <0.01
**DENV-4**	**Sub-district**	5	0.33 (0.15, 0.59)	0.63 (0.56, 0.72)	<0.01, <0.01
	**Village**	15	0.65 (0.47, 0.88)	0.83 (0.78, 0.89)	<0.01, <0.01
	**School**	9	0.59 (0.37, 0.87)	0.77 (0.68, 0.87)	<0.01, <0.01
	**Year**	4	0.19 (0.1, 0.38)	0.5 (0.43, 0.68)	<0.01, <0.01

Viruses isolated via school-based surveillance only. Traits showing significant phylogenetic structure are indicated in bold.

1 AI and PS scores are based on mean values representing the association between trait and phylogeny.

2 *P*-values are based on median values representing the association between trait and phylogeny.

An important observation from our study is the strength of temporal DENV clustering by year. This suggests that seasonal bottlenecks commonly occur in this population and, hence, that only a subset of viral genetic diversity within a given year survives into the next. This may in turn result in regular reductions in population immunity and competition among viruses, thus allowing the introduction and dissemination of viruses from outside of the study area. While persistence of viral lineages and *in situ* evolution occur over multiple seasons in some cases, these processes are relatively limited compared to that of viral migration (i.e. importation), which appears to occur via movement of infected people, play a dominant role in generating diversity within each serotype, and contribute to dynamics in patterns of DENV transmission. This finding is similar to that reported for Kamphaeng Phet in 2001 [13], and further indicates that it is important to account for the entry and re-entry of DENV lineages from external populations when considering the potential response to vaccination programs. In the context of forthcoming vaccine trials, and given the potential for imperfect vaccination and complex immune interactions, these results imply that the risk of DENV transmission and severe disease within a community will not only be determined by the vaccination levels within that geographic area, but also by the vaccination status and movement of people in neighboring regions.
